# Progress of CRISPR-Cas13 Mediated Live-Cell RNA Imaging and Detection of RNA-Protein Interactions

**DOI:** 10.3389/fcell.2022.866820

**Published:** 2022-03-09

**Authors:** Huake Cao, Yuechen Wang, Ning Zhang, Siyuan Xia, Pengfei Tian, Li Lu, Juan Du, Yinan Du

**Affiliations:** ^1^ School of Basic Medical Sciences, Anhui Medical University, Hefei, China; ^2^ First School of Clinical Medicine, Anhui Medical University, Hefei, China; ^3^ Second School of Clinical Medicine, Anhui Medical University, Hefei, China; ^4^ First Affiliated Hospital of Anhui Medical University, Hefei, China; ^5^ School of Medicine, The Chinese University of Hong Kong, Shenzhen, China; ^6^ Longgang District People’s Hospital of Shenzhen & The Second Affiliated Hospital, The Chinese University of Hong Kong, Shenzhen, China

**Keywords:** CRISPR, Cas13, RNA imaging, RNA-protein interactions, RNA targeting, RNA biology

## Abstract

Ribonucleic acid (RNA) and proteins play critical roles in gene expression and regulation. The relevant study increases the understanding of various life processes and contributes to the diagnosis and treatment of different diseases. RNA imaging and mapping RNA-protein interactions expand the understanding of RNA biology. However, the existing methods have some limitations. Recently, precise RNA targeting of CRISPR-Cas13 in cells has been reported, which is considered a new promising platform for RNA imaging in living cells and recognition of RNA-protein interactions. In this review, we first described the current findings on Cas13. Furthermore, we introduced current tools of RNA real-time imaging and mapping RNA-protein interactions and highlighted the latest advances in Cas13-mediated tools. Finally, we discussed the advantages and disadvantages of Cas13-based methods, providing a set of new ideas for the optimization of Cas13-mediated methods.

## 1 Introduction

CRISPR-Cas (Clustered Regularly Interspersed Short Palindromic Repeat-CRISPR-Associated) systems have received increasing attention in the scientific community due to their accurate targeting and excellent editing ability. The CRISPR-Cas loci encode an adaptive immune system in most archaea and many bacteria, which specifically recognize and cut foreign DNA or RNA sequences for improved protection against viral invaders (Barrangou et al., 2007; Marraffini and Sontheimer, 2010; Koonin and Makarova, 2013; Makarova et al., 2013; Barrangou and Marraffini, 2014).

CRISPR-Cas systems have two principal modules, the adaptive module and the effector module (Koonin et al., 2017). The adaptive module is basically composed of endonuclease Cas1 and structural subunit Cas2 (Amitai and Sorek, 2016; Koonin et al., 2017), whereas the effector module varies widely between CRISPR-Cas types and subtypes (Makarova et al., 2013; Amitai and Sorek, 2016; Mohanraju et al., 2016). Cas effectors consist of Class I and Class II (Murugan et al., 2017). The two classes are further divided into six types (I–VI). In Class I (Type I, III, and IV), the effector is composed of a large multi-subunit complex, whereas in Class II (Type II, V, and VI), the effector is a single-protein endonuclease (Makarova et al., 2015; Mohanraju et al., 2016; O’Connell, 2019). Although Class I is widely distributed in archaea and bacterial genomes (Jackson et al., 2017; Shmakov et al., 2017), many researchers prefer to use Class II due to its high efficiency with just one multidomain protein. Before Cas13 was discovered, DNA targeting was still the research tendency of methods based on CRISPR-Cas systems. In 2015, Shmakov et al. (2015) first reported the existence of Cas13 (formerly named C2c2). Cas13 has fascinating prospects due to its characteristic of specific RNA targeting. To date, Cas13 has been widely used in RNA detection, imaging, and manipulation of RNA biology (O’Connell, 2019).

RNA is an important component of gene expression and regulation and undergoes complex dynamic processes that influence gene expression in different ways (Braselmann et al., 2020). RNA imaging can be used to visualize gene expression and regulation. This strengthens the understanding of cell life activities and provides new methods and ideas for both the diagnosis and treatment of diseases (Darnell et al., 2010; Yamada et al., 2011; Chao et al., 2012; Ke et al., 2013; Lee et al., 2014; Buxbaum et al., 2015). Given that RNA does not fluoresce by itself, a variety of RNA imaging probes have been designed to achieve RNA imaging. However, each approach has limitations. CRISPR-Cas13 is a new tool in RNA imaging in living cells. For example, dCas13a (LwaCas13a) labeled with GFP can achieve imaging stress-induced bulk *β-actin* mRNA movement (Abudayyeh et al., 2017). With the discovery of more Cas13 proteins, some Cas13-mediated imaging tools have been created and attracted much attention.

The interactions of RNA and its binding proteins (RBPs) have been recognized as one of the real components that regulate cellular functions, such as precise translation of spatiotemporal localization and promotion of correct cell expression (Dreyfuss et al., 2002). RBPs play a vital role in RNA biology and disease treatments (Gebauer et al., 2021). Recently, due to the development of the CRISPR-Cas system, a new hypothesis of identifying RBPs has stood out. Based on the discovery of dCas13 by Zhang’s group*,* which retained the targeting potential of Cas13 and removed its cleavage ability (Abudayyeh et al., 2017), Cas13 can be used as bait to pull down RBPs. To date, Cas13-mediated methods such as CARPID and CRUIS have been developed, providing new ideas for the detection of RBPs (Yi et al., 2020; Zhang et al., 2020).

In this review, we first described the current findings on Cas13. Then, we discussed various Cas13-mediated imaging tools in the field of RNA imaging and detection of RNA-protein interactions and compared them with conventional tools. Finally, we comprehensively analyzed the advantages and disadvantages of Cas13-based imaging technology, provided relevant suggestions based on existing research, and put forward prospects for future research.

## 2 CRISPR-Cas13

Cas13 (type VI system) is the only member of the CRISPR-Cas systems that can specifically target RNA, in addition to the Class I Type III systems (Abudayyeh et al., 2016; Tamulaitis et al., 2017). Cas13 has two enzymatically active Higher Eukaryotes and Prokaryotes Nucleotide-binding (HEPN) RNase domains (Anantharaman et al., 2013). One RNase is responsible for crRNA preprocessing, helping to form a mature type VI interference complex, whereas the other one has two HEPN endoRNase domains that mediate the precise cleavage of RNA (Shmakov et al., 2015; Abudayyeh et al., 2016; East-Seletsky et al., 2017; Smargon et al., 2017; Konermann et al., 2018; Yan et al., 2018). Generally, the Cas13 protein families have been divided into four subtypes based on the phylogeny of their effector complexes, namely, Cas13a (previously known as C2c2), Cas13b, Cas13c, and Cas13d (Shmakov et al., 2017; Konermann et al., 2018; Yan et al., 2018). Recently, two new proteins of Cas13 (Cas13X and Cas13Y) have also been reported (Xu et al., 2021).

Cas13a is a large protein that belongs to the type VI-A CRISPR-Cas system (Shmakov et al., 2015). Cas13a contains the nuclease (NUC) lobe and crRNA recognition (REC) lobe. The NUC lobe consists of the HEPN-1 domain, HEPN-2 domain, Helical-2 domain, and Helical-3 domain. The REC lobe consists of an N-terminal domain (NTD), a Helical-1 domain, and a cleft accommodating the crRNA repeat region (Liu et al., 2017a; Liu et al., 2017b; Knott et al., 2017). At present, several Cas13 proteins have been found, such as LshCas13a, LbuCas13a and LwaCas13a (Abudayyeh et al., 2017; Liu et al., 2017a; Liu et al., 2017b). In addition to pre-crRNA processing, these Cas13 proteins also have highly different structures at the architectural and domain organization levels (Knott et al., 2017; Liu et al., 2017a; Liu et al., 2017b). Recent studies showed that LwaCas13a was the most effective protein among fifteen Cas13a orthologs with no significant PFS motif, which provides a potential platform to research RNA targeting (Abudayyeh et al., 2017).

Cas13b is more powerful than Cas13a. Zhang’s group tested 21 orthologs of Cas13a, 15 orthologs of Cas13b and seven orthologs of Cas13c for knockdown activity of Cas13 family members and indicated that the knockdown level of PspCas13b increased continuously relative to LwaCas13a (the average knockdown rate of PspCas13b was 92.3%, while that of LwaCas13a was 40.1%) (Cox et al., 2017). Compared with other Cas13 proteins, Cas13b has many structural differences. First, unlike Cas13a, Cas13c, and Cas13d, the HEPN domains of Cas13b are at the extreme N and C termini of the linear protein (Shmakov et al., 2017; Smargon et al., 2017). Second, the direct repeat of the Cas13b crRNA is at the 3′ end (Abudayyeh et al., 2016; Konermann et al., 2018; Yan et al., 2018). Third, the target RNA is allowed to access the opened central channel of PbuCas13b, whereas Cas13a and Cas13d require a shared solvent-exposed cleft that grasps the target RNA (Liu et al., 2017a; Zhang et al., 2018; Slaymaker et al., 2021).

The flexibility of Cas13d makes it more widely used because the enzyme is smaller than other Cas13 subtypes in terms of body size (Yan et al., 2018). One main reason is the absence of the Helical-1 domain compared to Cas13a (Liu et al., 2017b; Yan et al., 2018). Meanwhile, Cas13d lacks an appreciable sequence that constrains the target flanking sequences. In addition, Cas13d has a special mechanism for crRNA processing. Cas13d shows powerful collateral RNase activity and target cleavage (Yan et al., 2018) and is suitable for *in vivo* delivery because of its relatively small size (Abudayyeh et al., 2017).

Cas13X and Cas13Y are new family members of Cas13 which were identified from hypersaline samples. Cas13X can be divided into Cas13X.1 and Cas13X.2, while Cas13Y can be divided into Cas13Y.1 to Cas13Y.5. Compared with the conventional Cas13 protein, Cas13X.1 contains only 775 amino acids, which is the smallest Cas13 protein at present. Cas13X.1 was further truncated from 775 to 445 aa, which solves the delivery obstacles of various Cas13-based base editors *in vivo*. With the combination of ADAR2dd variants and truncated dCas13X.1, new editors used for A-to-I or C-to-U editing of various RNA loci in mammalian cells have been designed and show more advantages compared with REPAIR (Cox et al., 2017) systems and RESCUE systems (Abudayyeh et al., 2019; Xu et al., 2021).

With the further study of Cas13, Zhang’s group mutated conserved catalytic residues in the HEPN domains of Cas13, removing its nuclease activity (Cox et al., 2017). The mutant Cas13 is called CRISPR-catalytically dead CRISPR-Cas13 (dCas13). Due to a lack of cleavage ability, dCas13 becomes a great RNA binding platform for RNA modification, which is guided by gRNA to deliver engineering enzymes to the target region. On this basis, an increasing number of RNA tools based on dCas13 have been developed and widely applied in the fields of cell biology, disease and imaging (Tang et al., 2021).

## 3 Classic Imaging Methods and CRISPR/Cas13-Mediated Ribonucleic Acid Imaging

In the last dozen years, technological advances in RNA imaging in living cells have revolutionized cell biology. Many tools have been created and widely used for the recognition of numerous types of RNAs, including mRNAs and noncoding RNAs (Urbanek et al., 2014). RNA imaging probes can be classified into two categories: probes imaging exogenous RNAs and probes imaging endogenous RNAs, depending on whether the target RNA needs to be preprocessed. Recently, due to the excellent capability of RNA targeting, several attempts have been made to introduce new Cas13-mediated imaging methods. dLwaCas13a was first demonstrated to label abundant *β-actin* mRNA molecules after stress (Abudayyeh et al., 2017). Thereafter, a growing number of direct homologs of Cas13 proteins were reported to be applied to RNA imaging (Yang et al., 2019). In this section, we mainly focus on fluorescence-based CRISPR-Cas13 approaches and compare them with other imaging methods.

### 3.1 Classic Imaging Methods

#### 3.1.1 Probes Imaging Endogenous Ribonucleic Acids

Probes imaging exogenous RNAs in living cells mainly includes Molecular beacons (MBs), Nano-MBs, and Quencher-free probes. MBs are oligonucleotide probes formed by antisense stem-loop with a fluorophore and quenching agent at the end. The stem-loop structures contain recognition sequences binding to target RNA (Tyagi and Kramer, 1996; Xia et al., 2017). With a good fluorophore-quench pair, MB fluorescence can be increased by 20–100 times after hybridization with target RNA (Marras et al., 2002). However, MBs require carrier proteins to transport them into cells and hence cannot be directly used to image RNA in live-cells. In addition, the widespread use of MBs is limited by false-positive signals (FPS) due to their biological stability (Tyagi and Alsmadi, 2004; Chen et al., 2007).

As the quencher and the carrier, nanoparticles can combine with MBs to form Nano-MBs that consist of gold nanoparticles (GNPs) (Dubertret et al., 2001). GNP-Nano-MBs are the most typical Nano-MBs and can track the spatial distribution of target RNAs and quantify their expression (Briley et al., 2015). GNP-Nano-MBs have many advantages: a high signal-to-noise ratio (Dubertret et al., 2001), excellent resistance to enzyme degradation (Seferos et al., 2009), high cellular uptake (Choi et al., 2013), and a longer imaging fluorescence lifetime (Shi et al., 2016). However, GNP-Nano-MBs also contribute to RNA downregulation due to stable binding with targeted mRNA (Seferos et al., 2007; Prigodich et al., 2009).

Quencher-free probes can quench fluorophores by themselves, resulting in low background fluorescence without quenchers. Forced Intercalation Probes (FIT Probes) and Exciton-Controlled Hybridization-sensitive fluorescent Oligonucleotide probes (ECHO Probes) are two classical probes among them. Within the FIT probe, thiazole orange (TO) dyes serve as fluorescent base surrogates and signal hybridization in a single-nucleotide specific manner. The FIT probe can be used to develop brighter probes by incorporating fluorophore intercalators with higher fluorescence (Hövelmann et al., 2013). ECHO probes are sequence-specific and hybridization-sensitive probes that serve as artificial fluorescent nucleobases for RNA detection (Okamoto, 2011). However, ECHO probes are less robust and seldom used because the fluorescence increase strongly relies on the targeted RNA sequence (Okamoto, 2011).

#### 3.1.2 Probes Imaging Exogenous Ribonucleic Acids

Probes imaging exogenous RNAs in living cells mainly include the RNA binding protein-fluorescent protein system (RBP-FP system), bimolecular fluorescence complementation (BiFC) system, RNA aptamer/fluorophore system, and reporter gene system. The RBP-FP system can be applied in mRNA imaging. RBP refers to MS2 coat protein (MCP) binding to MBs (MS2-binding sequence), and FP refers to GFP. In this imaging system, the targeted mRNA is first pretreated by inserting six units of MBs into the 3′-UTR (untranslated region). Subsequently, the MCP-GFP fusion protein is added to the pretreated system, which helps illuminate the specific mRNAs. The RBP-FP system was first used in living yeast to track the transport and localization of *ASH1* mRNA particles (Bertrand et al., 1998). However, recent studies have shown that MS2 binding site arrays inhibit 5′ to 3′ degradation of mRNA. which results in the accumulation of 3′ mRNA fragments (Garcia and Parker, 2015, 2016; Heinrich et al., 2017). In addition, the accumulation of MBs fragments has a significant effect on mRNAs with high regulation and short life span (Tutucci et al., 2018). The analysis is often confounded by background fluorescence.

BiFC uses two different RBPs conjugated to two halves of split FPs (RBP1-N-FP and RBP2-C-FP) (Hu et al., 2002). MCP-PCP-based (Wu et al., 2014), PUMHD-based (Yoshimura and Ozawa, 2016), and aptamer-protein-based (Valencia-Burton and Broude, 2007) BiFC systems are among the main types of BiFC systems. This method is suitable to visualize long-lived RNAs, not for real-time visualization, because the fusion of N-FP and C-FP requires time and is irreversible (Wu et al., 2014).

Unlike RBP-FP systems and BiFC systems, RNA aptamer/fluorophore systems enable real-time imaging due to the fast-binding interactions of the aptamer and small molecule fluorophores (Bertrand et al., 1998; Paige et al., 2011; Wu et al., 2014). There are three advantages of the systems: 1) fast imaging speed (Paige et al., 2011), 2) low background noise (Song et al., 2014), and 3) resistance to photobleaching and suitability for long-term tracking of RNAs.

Reporter gene systems are specifically suitable for miRNA imaging (Kim S. et al., 2009; Niu and Chen, 2009; Niu and Chen, 2012). It can be used for imaging pri-miRNA (primary miRNA) cutting, pri-miRNA transcription, ds-miRNA (miRNA-miRNA*), and miRNA function; however, it mostly focuses on the imaging of mature miRNAs (Kim H. J. et al., 2009; Kim S. et al., 2009; Tu et al., 2014; Choi et al., 2016; Wang et al., 2016) (Table 1).

**TABLE 1:** Classical RNA imaging methods and Cas13-mediated methods.

Methods	Application	*In vitro*/*In vivo*	Advantages	Disadvantages	References
RBP-FP system	mRNA	*In vitro*	High resolution	High background signals	Zimyanin et al. (2008), van Gemert et al. (2009), Lionnet et al. (2011), Muramoto et al. (2012), Yoshimura et al. (2012), Hocine et al. (2013), Hayashi et al. (2014), Rino et al. (2014)
BiFC system	mRNA	*In vitro*	Low background signals	Irreversibility; only suitable for visualizing long-lived RNAs; not suitable for visualizing real-time	Shyu and Hu (2008), Yamada et al. (2011)
RNA aptamer/fluorophore system	5S RNAs, 6S RNAs, mRNA	*In vitro*	Fast imaging; real-time imaging; suitable for long-time tracking of RNAs	High background signals	Paige et al. (2011), Dolgosheina et al. (2014), Shin et al. (2014)
Reporter gene system	miRNA	*In vitro*	Without affecting the properties of RNAs	Only suitable for the visualization of miRNA	Kim H. J. et al. (2009), Ko et al. (2009), Kang et al. (2012), Wang F. et al. (2013)
MBs	miRNA, mRNA	*In vivo*	Wide application	False-positive signals	Kang et al. (2011), Wang Z. et al. (2013), Lee et al. (2015b), Tay et al. (2015); Xia et al. (2017)
Nano-MBs	mRNA, miRNA	*In vivo*	Low background signals; excellent resistance to enzyme degradation; high cellular uptake; longer imaging fluorescence lifetime	RNA downregulation	Riahi et al. (2014), Lee et al. (2015a), Wang et al. (2015)
Quencher-free probes	mRNA, 28S rRNA, snoRNA, polyA RNA	*In vivo*	Robust; high sensitivity and specificity	Easily subject to self-dimerization	Kummer et al. (2011), Okamoto (2011), Oomoto et al. (2015)
dCas13a-NF	mRNA	*In vivo*	High efficiency; robust; low background noise; real-time imaging	Cumbersome design	Abudayyeh et al. (2017)
CRISPR-dPspCas13b-mediated imaging	lncRNA, mRNA	*In vivo*			Yang et al. (2019)
Imaging using dCas13 and dCas9	DNA, mRNA	*In vivo*			Wang et al. (2019), Yang et al. (2019)

### 3.2 Methods Based on Cas13

#### 3.2.1 dCas13a-NF

**FIGURE 1:**
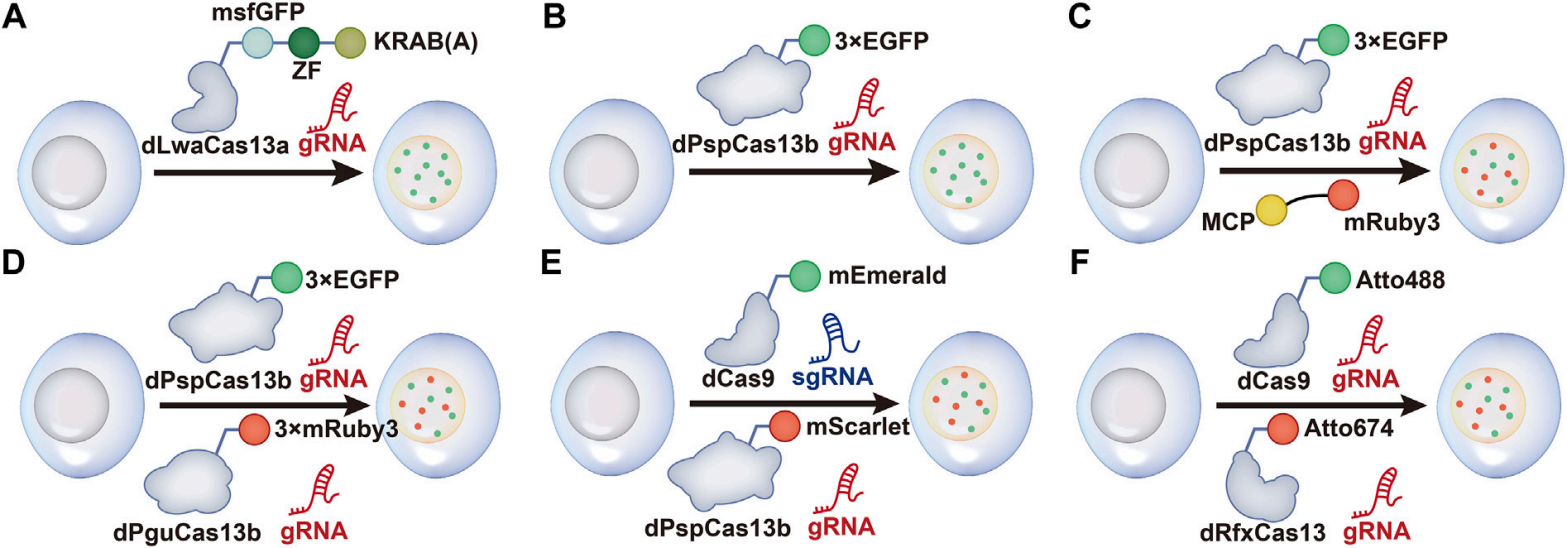
Schematic of CRISPR/Cas13-mediated RNA imaging methods. **(A)** dCas13a-GFP-KRAB construction for negative-feedback imaging. The dLwaCas13a incorporates a negative-feedback system based upon zinc finger self-targeting and the KRAB repression domain to image ACTB mRNA. **(B)** dPspCas13b-3 × EGFP labeling system. dPspCas13b is tagged with several green fluorescent proteins EGFP to image mRNAs. **(C)** Dual-color RNA labeling using a combination of the dPspCas13b and MS2- MCP systems. A total of 24 × MS2 (24 copies of the MS2 stem loop)-NEAT1-KI HeLa cells are constructed. Then, RNAs are labeled simultaneously by transfecting dPspCas13b-3 × EGFP, gRNAs for NEAT1, and MCP-mRuby3 into the cells. **(D)** Dual-color RNA labeling by different dCas13b systems. RNAs are labeled with dPspCas13b and dPguCas13b in HeLa cells. **(E)** Dual-color labeling using dCas9 and dPspCas13b. DNA and RNAs are labeled simultaneously in living cells by combining the dCas9-mEmerald and dPspCas13b-mScarlet systems. **(F)** Dual-Color labeling using dCas9 and dRfxCas13. Atto488-labeled dCas9 and Atto647-labeled dRfxCas13 are used to image genomic DNA and RNA transcripts.

Cas13a was previously used for RNA knockdown and binding. Recently, a method called dCas13a-NF was created by Zhang’s group to image RNA. First, the investigators evaluated 15 orthologs and identified LwaCas13a as the most effective Cas13a that is highly active and lacks PFS in bacteria. dCas13a was generated by inactivating catalytic arginine residues, and a significant enrichment of the corresponding target was presented by pulldown of dCas13a. To reduce background noise resulting from free proteins, a negative-feedback system was then designed based on zinc finger self-targeting and KRAB domain repression. The dCas13a (LwaCas13a) labeled with GFP can effectively re-localize and achieve imaging of *β-actin* mRNA under stress (Abudayyeh et al., 2017) (Figure 1A).

#### 3.2.2 CRISPR-dPspCas13b-Mediated Imaging

In a separate study, Yang et al. used the co-localization of *NEAT1* to screen eight fluorescent protein-labeled dCas13 direct homologs and thus explored their RNA imaging ability. *NEAT1* is a moderately expressed long noncoding RNA (lncRNA), and the co-location of multiple NEAT1 molecules on the fluorescence background shows sufficient S/N (signal-to-noise) differentiation, which is suitable for screening RNA imaging. The results showed that dPspCas13b and dPguCas13b were the most effective Cas proteins. However, the mentioned dLwaCas13a protein is not available for *NEAT1*. Previous studies have discovered that the length and mismatch location of gRNA affected the efficiency of RNA cleavage and labeling (Abudayyeh et al., 2017; Cox et al., 2017).

For RNA imaging, length and targeting position are crucial to the gRNA of dPspCas13b (Yang et al., 2019). It is noteworthy that the length of gRNA is preferably within 20–27 nt, whereas for RNA cleavage, the suitable length is 30 nt. In addition, the investigators assessed RNA-binding specificity. The results indicated that the binding activity of dPspCas13b was more sensitive to mismatches in the middle and direct repeat regions. For example, the mismatches at positions 17 and 18 could lead to the disability of labeling. Thereafter, the optimal gRNA was screened prior to labeling *MUC4* mRNA and *GCN4* using the dPspCas13b system (Figure 1B). Notably, dRfxCas13d cannot be used due to the generation of abnormal signals (Yang et al., 2019). smFISH further confirmed the efficiency and accuracy of the labeling mRNAs containing repeated sequences in the nucleus and cytoplasm. Compared to the classic MS2-MCP system, the labeling efficiency of CRISPR-dPspCas13b reached approximately 80%, whereas the MS2-MCP system was only 30% (Figure 1C). Importantly, this system can achieve dual-color imaging for single RNAs and image two different RNAs using two orthogonal CRISPR-dCas13 proteins (Figure 1D). However, the steps of pretreatment are tedious and hence require optimization.

#### 3.2.3 Imaging Using dCas13 and dCas9

Previous studies have also shown that fluorescently labeled nuclease-deficient Cas9 (dCas9) protein is excellent at DNA or RNA imaging (Jolly et al., 2002; Chen et al., 2013). Furthermore, it has been reported that the dCas9 protein was fused with an enhanced green fluorescent protein (EGFP) to enrich the fluorescence signal at the target genome site for imaging (Chen et al., 2013). Therefore, the combination of dCas9 and dCas13 provides a new idea for the simultaneous imaging of RNA and DNA in living cells.

Research showed that after stress treatment of cells, dCas9 and dCas13 (dPspCas13b) could realize dual-color imaging of DNA and RNA (Figure 1E). dCas9 successfully labeled *MUC4* and *SatIII* DNAs, and dPspCas13b-mScarlet successfully labeled *MUC4* and *SatIII* RNAs, realizing simultaneous labeling of genomic DNA and transcriptional RNAs (Yang et al., 2019). In addition, Wang et al. (2019) developed a robust, versatile approach named CRISPR live-cell fluorescent (LiveFISH) *in situ* hybridization using fluorescent oligos, which is suitable for genome tracking in broad cell types. They used another Cas13 protein (RfxCas13d) to bind dCas9, visualizing both the MS2-repeat-tagged mRNA and DNA in real time (Figure 1F).

## 4 CRISPR/Cas13-Mediated Ribonucleic Acid-Protein Interactions Detection

Revealing the interactions of RNA and RBPs offers essential clues for understanding RNA biology. The interactions are complex, and a particular RNA tends to have fixed protein-binding domains with multiple RBPs. Similarly, a particular protein can also work with different RNAs (Weissinger et al., 2021). When the RNA-RBP interaction is disturbed, it will inevitably lead to many diseases, such as cancer, neurogenic diseases, and leukemia (Tolino et al., 2012; Pereira et al., 2017; Elcheva and Spiegelman, 2021).

Currently, there are a variety of methods for identifying RNA and its binding proteins, which can be broadly divided into two categories, protein-centered and RNA-centered (Ramanathan et al., 2019). Protein-centered methods are used to select a specific protein and observe the RNAs bound to it, such as RNA coimmunoprecipitation (RIP) and cross-linking immunoprecipitation (CLIP) (Gagliardi and Matarazzo, 2016; Lee and Ule, 2018). Furthermore, CLIP has been optimized with the development of mass spectrometry and sequencing technology (Licatalosi et al., 2008). The RNA-centered methods are for selecting a specific RNA and identifying the respective RBPs, such as RNA hybridization capture, RNA tagging based on gene aptamers, and proximity labeling (Tsai et al., 2011; West et al., 2014; Ramanathan et al., 2018). However, these methods have similar disadvantages, including the need for specific antibodies and strict elution conditions. The study of dCas13 provides a new idea for imaging, and some Cas13-mediated RNA-centered methods have been reported. In this section, we highlight Cas13-mediated methods and explore existing methods regarding their advantages and limitations.

### 4.1 Common Ribonucleic Acid-Centered Methods

RNA-centered methods can roughly be divided into *in vitro* and *in vivo* methods (Moore, 2005). *In vitro* methods usually use *in vitro*-transcribed (IVT) RNA with specific markers and then add cell extracts and elution proteins and analysis thereafter (Leppek and Stoecklin, 2014; Zheng et al., 2016). However, *in vitro* methods tend to ignore the influence of the intracellular environment on protein interactions. Moreover, the RNA transcribed *in vitro* may be different from the actual RNA in cells in terms of structure and morphology (Faoro and Ataide, 2014).

*In vivo*, endogenous RNA-protein imaging can overcome the described shortcomings. Endogenous methods can be divided into two categories: methods that use protein-RNA cross-linking and do not require protein-RNA cross-linking. The cross-linking methods first use formaldehyde or UV to cross-link the RBPs and RNA into a reversible or irreversible covalent bond, and then the protein is pulled down using a specific probe for elution (Sutherland et al., 2008; Li et al., 2014). Common cross-linking methods include RNA Antisense Purification (RAP), tandem RNA Isolation procedure (TRIP), and peptide nucleic acid (PNA)-assisted identification of RBPs (PAIR) (Zeng et al., 2006; Matia-González et al., 2017; McHugh and Guttman, 2018). Different from the previous cross-linking method, chromatin isolation by RNA purification (ChIRP) and capture hybridization analysis of RNA targets (CHART) use formaldehyde to cross-link RNA to proteins (Chu et al., 2011; Simon et al., 2011) (Table 2).

**TABLE 2:** RNA-content methods: common methods and methods based on Cas13.

Methods	Application	*In vitro*/*In vivo*	Advantages	Disadvantages	References
Biotinylated RNA	mRNA	*In vitro*	Strong combination between streptavidin beads and biotinylated RNA	Potentially biased toward abundant proteins	Zheng et al. (2016)
S1 aptamer	mRNA	*In vitro*	Simple purification without the need for recombinant protein production	Potentially biased toward abundant proteins; interference with native RBPs formation; unspecific interactions	Leppek and Stoecklin (2014)
RAP	lncRNA	*In vivo*	Strong combination between probe and RNA	High input cell numbers	McHugh et al. (2015)
TRIP	mRNA	*In vivo*	No need of genetic manipulation; UV cross-linking	Careful design and evaluation of ASO; differences in ASO binding sites may reduce efficiency	Matia-González et al. (2017)
PAIR	mRNA	*In vivo*	UV cross-linking	Difficult to product peptide nucleic acid	Zeng et al. (2006)
CHART	lncRNA	*In vivo*	Simple design; split pools of tiling oligonucleotide probes and glutaraldehyde crosslinking ensure the success	High input cell numbers	Chu et al. (2011)
RaPID	mRNA	*In vivo*	Low number of cells needed; interrogate motifs <50 nucleotides	Requires BoxB link to RNA; not all proteins can be detected due to biotinylation; it’s difficult to tell whether the protein is acting directly or indirectly	Ramanathan et al. (2018)
CARPID	lncRNA	*In vivo*	No need of genetic manipulation; Multiple gRNAs are designed to reduce background noise	Need a high abundance of targeted RNA; unstable binding; difficult to detect all the proteins due to the limitation of gRNA	Yi et al. (2020)
Cas13-based APEX targeting	hTR	*In vivo*	No need of genetic manipulation; introduce double-stranded RNA binding domain (dsRBD) to improve the stability of dCas13 complex	Need a high abundance of targeted RNA; unstable binding; difficult to detect all the proteins due to the limitation of gRNA	Han et al. (2020)
CRUIS	lncRNA, mRNA	*In vivo*	No need of genetic manipulation; no restriction on the type of RNA	Need a high abundance of targeted RNA; unstable binding; difficult to detect all the proteins due to the limitation of gRNA	Zhang et al. (2020)
CBRPP	lncRNA, mRNA	*In vivo*	No need of genetic manipulation; no restriction on the type of RNA	Need a high abundance of targeted RNA; unstable binding; difficult to detect all the proteins due to the limitation of gRNA	Li et al. (2021)
RPL	snRNA	*In vivo*	No need of genetic manipulation; no restriction on the type of RNA	Need a high abundance of targeted RNA; unstable binding; difficult to detect all the proteins due to the limitation of gRNA	Lin et al. (2021)
CBRIP	snRNA	*In vivo*	No restriction on the type of RNA; high stability and specificity	Need a high abundance of targeted RNA; difficult to detect all the proteins because of the limitation of gRNA	Chen et al. (2021)

Recently, a proximity labeling technique has been used to map molecular interactions in living cells (Roux et al., 2012). The technique does not require protein-RNA cross-linking, which uses biotin ligases such as BirA* and BioID2 or ascorbate peroxidases such as APEX and APEX2 to produce biotin around itself, which can mark surrounding proteins for subsequent purification.

The aptamer technique combined with the proximity labeling technique forms a mature technique for mapping RBPs. For example, RNA-protein interaction detection (RaPID) binds biotin ligase to BoxB, which helps deliver the system around the targeted RNA through the *λ* -n domain, and the surrounding proteins are hence labeled (Ramanathan et al., 2018). Other aptamers, such as MS2, have also been used to investigate RNA-protein interactions combined with ascorbate peroxidase (Han et al., 2020). However, this approach is limited by complex artificial design and biological expression. In addition, with the continuous development of CRISPR systems, dCas13 can directly target RNA of interest at the endogenous level without adaptor fusion (Abudayyeh et al., 2017). Therefore, Cas13-mediated methods are gradually becoming popular in the scientific community.

### 4.2 Methods Based on Cas13

#### 4.2.1 CARPID

**FIGURE 2:**
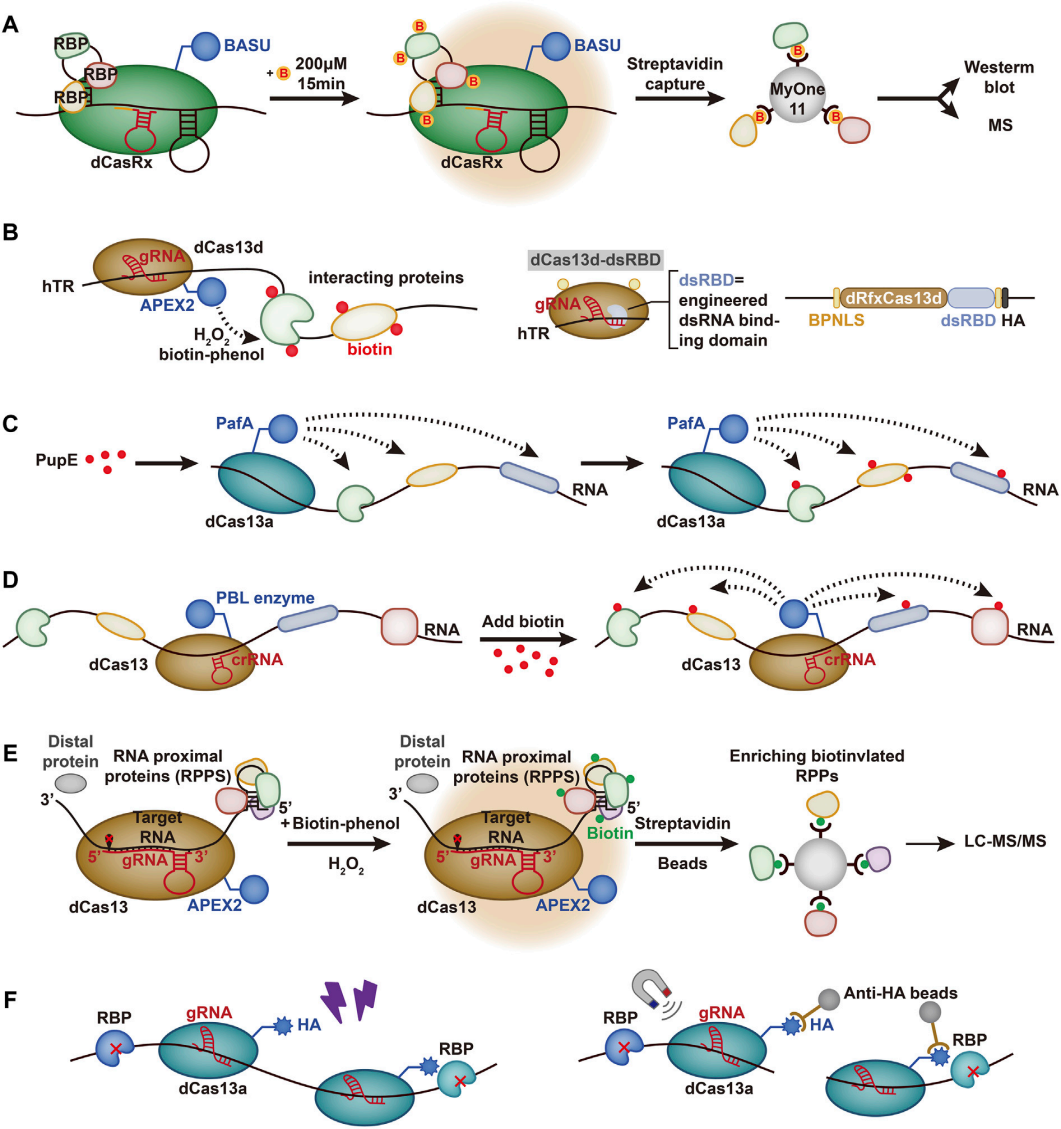
Schematic representation of RNA-contented methods based on Cas13. **(A)** CARPID. dCasRx is fused with BASU. Biotin is represented by yellow circles marked with red “B.” CARPID is directed by dCasRx to target the RNA of interest, and the RBPs are biotinylated. **(B)** RNA–protein interaction mapping via Cas13-based APEX targeting. APEX2 is fused with dCas13d and targeted to the hTR with the help of gRNA. H_2_O_2_ is added to cells preloaded with biotin-phenol, which is oxidized by APEX2 to phenoxy and covalently labels proximal endogenous proteins. A sequence-independent double-stranded RNA binding domain (dsRBD) from human protein kinase R (PKR) is fused to the C-terminus of the dRfxCas13d protein to strengthen the stability and targeting ability. A bipartite nuclear localization sequence (BPNLS) is used to optimize nuclear localization. **(C)** CRUIS. PafA is fused with dCas13a to target the RNA of interest and modifies the surrounding proteins by mediating PupE. **(D)** CBRPP. PBL is fused with dCas13 and targets specific RNA to covalently tag the surrounding protein. **(E)** RPL. APEX2 is fused with dCas13 and biotinylates RNA proximal proteins with H_2_O_2_ and biotin-phenol mediated by gRNA. The biotinylated proteins are enriched by streptavidin beads and analyzed by liquid chromatography-tandem mass spectrometry (LC-MS/MS). **(F)** CBRIP. dCas13a is fused with an HA tag and targets a specific RNA. RNA-protein interactions are stabilized by UV crosslinking, and the complexes are enriched by anti-HA beads.

*Xist* lncRNAs have been identified in mammals and play an important role in gene silencing (Wutz, 2011). In a study conducted by Yi et al. (2020) a system called the CRISPR-assisted RNA-protein interaction detection method (CARPID) was developed to identify RNPs based on CRISPR/Cas13. The CARPID specifically binds the fused BASU biotin ligase to the target lncRNA in living cells and then biotinylates the adjacent proteins and facilitates streptomycin affinity coupling magnetic bead separation (Figure 2A). Furthermore, the isolated proteins were quantitatively analyzed by mass spectrometry. A gRNA array consisting of two gRNA sequences is designed to reduce background noise and enhance the specificity of targeting. To ensure the optimal reaction conditions, experiments such as changing the induction time or changing the location of the biotin ligase are needed.

In addition, Yi et al. (2020) demonstrated the high specificity and repeatability of this method. The investigators used different gRNAs to form the experimental group and replicated the experiment in each group. The results showed that at least 447 proteins were shared between the replication groups, and unexpected RBPs, such as TAF15, were found. However, some proteins still fail to pass the detection threshold due to unstable binding between proteins and RNA. Furthermore, this method is still limited by the mediation of biotin ligase, and it is still difficult to operate low abundance RNA. The method requires improvement in future studies.

#### 4.2.2 Cas13-Based APEX Targeting

A new system to study human telomerase RNA (hTR) was developed by Han et al. for the detection of RNA-protein interactions. hTR plays a critical role in regulating cellular senescence as a template for reverse transcription and affects the development of diseases (Chen and Greider, 2004; Theimer and Feigon, 2006). In their study, Han et al. combined RfxCas13d with APEX2 (Konermann et al., 2018) to identify the RBPs of hTR (Theimer and Feigon, 2006; Han et al., 2020) (Figure 2B). They envisioned using dCas13 to send APEX2 near the targeted RBPs for labeling. APEX2 can use hydrogen peroxide (H_2_O_2_) as an oxidant to catalyze the one-electron oxidation of biotin-phenol (BP), which helps to pull down the proteins (Hung et al., 2016). However, although RfxCas13d was found to be effective in targeting and cutting hTR, there was no significant enrichment of gRNA co-expressed with dRfxCas13d, which was also demonstrated in other experiments (Li et al., 2021). Therefore, the combination of dRfxCas13d and hTR needs to be further optimized.

To improve the stability and targeting potential of the imaging system, Han et al. introduced a double-stranded RNA binding domain (dsRBD) (Masliah et al., 2013), which was fused to the C-terminus of the dRfxCas13d protein. The results showed that dCas13d-dsRBD-APEX2 can effectively improve the enrichment level (Konermann et al., 2018). However, some limitations still remain. For instance, the results from the CRISPR-based imaging system only partially overlapped with MCP-APEX2, which reflected that background noise was still present (Konermann et al., 2018).

#### 4.2.3 CRISPR-Based RNA-United Interacting System

Based on the development of the PUP-IT system for detecting membrane protein interactions (Liu et al., 2018), Zhang et al. (2020) created the CRISPR-based RNA-United Interacting System (CRUIS) by combining the PUP-IT system with dLwaCas13a (Figure 2C). The dLwaCas13a is used as a tracking tool to target specific RNA, and PUP-IT pulls down the proteins (Abudayyeh et al., 2017). To demonstrate the functioning of the system, Zhang et al. tested RBPs of NORAD (noncoding RNA activated by DNA damage) with CRUIS (Lee et al., 2016; Munschauer et al., 2018). The mass spectrometry results showed that CRUIS performed well in detecting validated RBPs, such as KHSPR and SRSF9, and could also detect unexpected proteins that were not considered RBPs (Munschauer et al., 2018; Zhang et al., 2020).

In addition to lncRNA, Zhang et al. (2020) also used this system to detect *p21* mRNA and detected hnRNA-bound proteins such as HNRNPK and HNRNPA1, which also indirectly reflected the maturation mechanism of mRNA. The results indicate that the CRUIS system has a wide range of applications and broad potential. However, the efficiency of single sgRNA protein detection is limited and thus greatly affects the detection efficiency. Moreover, the presence of RNA secondary structures also continues to influence the system (Zhang et al., 2020).

#### 4.2.4 CBRPP

Li et al. (2021) developed a set of systems named CBRPP based on the CRISPR system and PBL fusion selected by three experiments (Li et al., 2021). First, they fused dRfxCas13d and APEX2 to detect the RBPs of ACTB (Lam et al., 2015; Konermann et al., 2018) (Figure 2D). However, non-specific enrichment was found to be displayed regardless of whether the RfxcrRNA, the targeting sequence of ACTB, was transfected, indicating that dRfxCas13d-APEX2-NLS could not bind to the target RNA, which was also proven in the experiment conducted by Yang et al. (2019), Li et al. (2021). They then transfected dPspCas13b-APEX2-NES to detect *ACTB* mRNA and found the same inefficiency, which could be related to the high expression of dPspCas13b-APEX2-NES (Li et al., 2021). Later, they chose to fuse dPspCas13b with BioID2. The obtained data showed that the dPspCas13b-BioID2-NES system successfully recognized its interacting proteins. In addition, BioID2 can also help to identify transient proteins, and the targeted proteins can accumulate during reimaging, which can reduce the background noise (Li et al., 2021). Li et al. also used this system to detect NORAD in lncRNAs, showing good imaging performance. These findings revealed the strong potential of CBRPP in the imaging system (Ventura, 2016).

#### 4.2.5 RNA Proximity Labeling

Lin et al. (2021) developed a method known as RNA proximity labeling (RPL) based on the fusion of dPspCas13b with the adjacent marker enzyme APEX2, which can lead to biotinylation of proteins within a distance of 25 nm from the targeted RNA by inference (Figure 2E). Cas13b was selected for its high specificity and low miss efficiency, and APEX2 was selected for its high kinetic effects (Lam et al., 2015; Yang et al., 2019). The RPL system was used to analyze the nuclear RNA (ncRNA) *U1* and poly(A) tail proximal proteins and can rapidly identify RBPs and uncover novel RBPs such as KPNB1 (Lin et al., 2021). To reduce the background noise of the system, Lin et al. also took some measures, such as selecting the *U1* with high abundance (Stark et al., 2001) and designing three kinds of gRNA to be expressed in separate cell lines in combination with different regions. Importantly, the RPL system does not use UV cross-linking or genetic system-based operations, which can effectively avoid the biases of UV cross-linking and genetic interference with RNA function (Baranello et al., 2016; Laprade et al., 2020).

#### 4.2.6 CRISPR-Based RNA Interaction Proteomics

Recently, Chen et al. (2021) developed CRISPR-based RNA interaction proteomics (CBRIP) by combining dLwaCas13a with HA Tag (Figure 2F). Specific RNA is tracked using dCas13, and RBPs and RNA are then cross-linked using UV light. Finally, they are captured by anti-HA beads. The corresponding protein can be detected through mass spectrometry thereafter. Chen et al. proposed that limiting the expression of dCas13 was critical to improving the SNR. Therefore, Tet-on was designed as the promoter of dCas13 based on Dox, and gRNA was continuously induced by the *U6* promoter (Han et al., 2020; Chen et al., 2021). A total of 226 proteins were identified as RBPs of *U1* snRNA, and RPL7 was verified as a novel U1 binding protein. Meanwhile, the potential of this system for lncRNA was also demonstrated by validating RBM15 as an XIST-binding protein (McHugh et al., 2015). However, the whole CBRIP experiment requires large input cell numbers for maintenance of the capturing efficiency due to UV cross-linking (Chen and Greider, 2004).

## 5 Conclusion and Challenges

The precise targeting and editable properties of Cas13 make it an excellent tool for RNA imaging and detection of RNA-protein interactions. However, Cas13-mediated RNA imaging and mapping RNA-protein interactions are still in the exploration stage. Compared with other imaging methods, Cas13-mediated tools provide real-time imaging in living cells with lower background noise, better stability, and better imaging efficiency. Although these Cas13-mediated imaging methods have been optimized, there is still room for further improvements in their performance. Screening of Cas13 proteins is still tedious work, and not all proteins can be used for imaging. One reason could be that some Cas13 proteins cannot produce labeling signals. Furthermore, the design process of gRNAs is very difficult, and not all gRNAs can work well. Even small changes in gRNA position will lead to significant differences in imaging effects. In addition, the existing Cas13 imaging methods are only suitable for high abundance RNA whereas they cannot achieve good imaging effect for RNA with low abundance. Moreover, some cells also need to be processed in advance before imaging. For example, *SatIII* RNA visualization was achieved only after treatment with heat shock or SA (Yang et al., 2019). The mechanism and realization of this technology still require further exploration.

To date, multiple Cas13-mediated RNA-protein detection tools have been reported and initially evaluated. Cas-mediated methods have many advantages. It is noteworthy that Cas13 does not require pre-labeling of target RNA (Zheng et al., 2016), design of antisense probes (McHugh and Guttman, 2018) or insertion of MS2 or BoxB (Ramanathan et al., 2018; Mukherjee et al., 2019), which simplifies the experimental workflow. In addition, the imaging process is performed at the level of endogenous expression and does not involve any genetic manipulation, and the entire imaging process occurs in the living cell, which ensures that the detected interaction is closer to the real intracellular environment. However, the efficiency and specificity of targeting depend on the types of gRNA and Cas proteins, which requires rigorous and complicated screening. A larger Cas13 protein can affect the efficiency, while whether some newfound smaller Cas13 proteins present high efficiency is still questionable. Moreover, the expression level of the Cas13 protein complex in cells also affects the signal-to-noise ratio of the whole experiment (Han et al., 2020; Zhang et al., 2020; Li et al., 2021). One reason might be that the highly expressed Cas13 protein complex leads to a higher rate of labeling for nonspecific proteins. Therefore, inducing the low expression of Cas13 is an important part of the whole imaging system. In addition, the binding of gRNA to target RNA may be competitive with the binding of RBPs, while a single gRNA may reduce the RBP detection rate (Lin et al., 2021). Therefore, a RNA array can be set up as described by Yi et al. (2020) in CARPID. Furthermore, groups of different gRNAs can also be set up to reduce background noise (Lin et al., 2021). Meanwhile, the type of biotin also affects the efficiency of imaging (Li et al., 2021). The labeling of biotin is nonspecific; thus, all proteins within the range may be labeled, which may also cause damage to the binding of RNA and protein.

There are several directions for future optimization of the current approaches for Cas13-mediated RNA imaging and detection of RNA-protein interactions. First, a structural design database should be set up, and the design methods of gRNA should be optimized to simplify the design process of gRNA. With the development of deep learning technology, new gRNA efficiency prediction and design tools are expected to be developed. Second, more Cas13 proteins, especially newfound ones, can be screened out to further improve the targeting ability. Third, current linker proteins can be chemically modified so that they have a stronger signal when they bind to their target. Finally, optimizing the observation imaging equipment can also achieve better visualization results.

However, there are more difficult problems to address. The space structure of RNA changes may affect the targeted efficiency, such as the existence of the secondary structure, which may affect the gRNA combined with the target RNA (Zhang et al., 2020). In addition, the current imaging systems based on Cas13 still target high abundance RNAs, which is still a challenge for low abundance RNA, and the imaging efficiency still needs further increased (Han et al., 2020; Lin et al., 2021). In conclusion, Cas13-mediated methods for RNA imaging and detection of RNA-protein interactions are novel techniques. There is a need for further research to obtain solutions to the described challenges of imaging technology optimization based on the CRISPR system. In the near future, we could expect more reports on the improvement or development of Cas13-mediated methods.
